# ROS-Mediated Mitochondrial Pathway is Required for* Manilkara Zapota* (L.) P. Royen Leaf Methanol Extract Inducing Apoptosis in the Modulation of Caspase Activation and EGFR/NF-*κ*B Activities of HeLa Human Cervical Cancer Cells

**DOI:** 10.1155/2018/6578648

**Published:** 2018-06-06

**Authors:** Bee Ling Tan, Mohd Esa Norhaizan, Lee Chin Chan

**Affiliations:** ^1^Department of Nutrition and Dietetics, Faculty of Medicine and Health Sciences, Universiti Putra Malaysia, 43400 Serdang, Selangor, Malaysia; ^2^Laboratory of Molecular Biomedicine, Institute of Bioscience, Universiti Putra Malaysia, 43400 Serdang, Selangor, Malaysia; ^3^Research Centre of Excellent, Nutrition and Non-Communicable Diseases (NNCD), Faculty of Medicine and Health Sciences, Universiti Putra Malaysia, 43400 Serdang, Selangor, Malaysia; ^4^Department of Microbiology, Faculty of Biotechnology and Biomolecular Sciences, Universiti Putra Malaysia, 43400 Serdang, Selangor, Malaysia

## Abstract

*Manilkara zapota *(L.) P. Royen (family:* Sapotaceae*) is commonly called sapodilla, or locally known as* ciku*. The detailed mechanisms underlying* Manilkara zapota *leaf methanol extract against HeLa human cervical cancer cells have yet to be investigated. Therefore, our present study is designed to investigate the ability to induce apoptosis and the underlying mechanisms of* Manilkara zapota* leaf methanol extract inducing cytotoxicity in HeLa cells. The apoptotic cell death was assessed using Annexin V-propidium iodide staining. Intracellular reactive oxygen species (ROS) and mitochondrial membrane potential activities were measured using dichlorodihydrofluorescein diacetate and MitoLite Orange, respectively, by NovoCyte Flow Cytometer. Bax and Bcl-2 expression were evaluated using Enzyme-Linked Immunosorbent Assay. Caspase-3 activity was determined using a colorimetric assay. The associated biological interaction pathways were evaluated using quantitative real-time PCR. Our data showed that HeLa cells were relatively more sensitive to* Manilkara zapota* leaf methanol extract than other cancer cell lines studied. Overall analyses revealed that* Manilkara zapota *leaf methanol extract can inhibit the viability of HeLa cells, induce mitochondrial ROS generation, and inhibit nuclear factor-kappa B (NF-*κ*B) and epidermal growth factor receptor (*EGFR*) transcriptional activities. Our results suggested that* Manilkara zapota *leaf methanol extract might represent a potential anticervical cancer agent.

## 1. Introduction

Cervical cancer has become the fourth leading cancer in women, contributing to approximately 530,000 new cases in 2012, and represents 7.9% of all female cancers. Nearly 90% of the 270,000 deaths from cervical cancer occurred in low- and middle-income countries in 2015 worldwide [1]. Although locally advanced and early-stage diseases can be cured by chemoradiotherapy and radical surgery [2], conventional therapy is not likely effective due to undesirable effects, thereby limiting their use in many patients.

Epidermal growth factor receptor (EGFR) has emerged as a crucial therapeutic target in more than 30% of solid tumors, which is commonly associated with a poor prognosis [3]. Apart from EGFR signaling, nuclear factor-kappa B (NF-*κ*B) is a crucial transcription factor that has an ability to activate the large array of inflammatory mediators and has been recognized as a central player for cervical cancer [4]. Not only does NF-*κ*B activity stimulate tumor cell proliferation and inhibit apoptosis, but it also facilitates metastasis [4]. Given the pivotal role of NF-*κ*B in human cancer initiation, development, and metastasis, NF-*κ*B pathway may serve as a promising therapeutic target.

There have been extensive studies on natural compounds which showed a potent antiproliferative activity, in conjunction with having good antioxidant activities [5, 6]. Many plants especially traditional medicinal plants have been widely investigated for their antioxidant activity in the last few decades [7]. Emerging evidence has demonstrated the role of natural antioxidant in the prevention of chronic diseases including cancer and inflammation [8–10]. In continuation of the efforts towards the discovery of better treatment strategies for cervical cancer, plants have gained remarkable interest as an effective anticancer agent. In line with this, there is an unmet need to discover new anticancer agent with high efficacy and specificity but showing minimal adverse outcome.

*Manilkara zapota *(L.) P. Royen (family:* Sapotaceae*), commonly called sapodilla, or locally known as* ciku*, is an evergreen tree grown abundantly throughout India subcontinent including Bangladesh [11], though it is native to Central America and Mexico.* Manilkara zapota* leaf has been traditionally used for the treatment of diarrhea, cold, and coughs [12]. Nonetheless, there is no pharmacological study on anticervical cancer properties of* Manilkara zapota *leaf methanol extract in the literature. Our earlier study demonstrated that* Manilkara zapota *leaf water extract has cytotoxic activity towards human hepatocellular carcinoma (HepG2) and human colorectal adenocarcinoma (HT-29) cell lines (unpublished data). Therefore, this study was designed to investigate the ability to induce apoptosis and the underlying mechanisms of* Manilkara zapota* leaf methanol extract inducing cytotoxicity in HeLa cells. These molecular interactions underlying the apoptotic mediated signaling pathway in cellular function may be involved in the modulation of cervical cancer and deserve further elucidation.

## 2. Materials And Methods

### 2.1. Chemicals and Reagents

RPMI-1640 medium, Mycoplex™ fetal bovine serum (FBS), penicillin and streptomycin (100×), Dulbecco's Modified Eagle Medium (DMEM), and trypsin-ethylenediaminetetraacetic acid (EDTA) (1×) were bought from Gibco (Grand Island, NY, USA). Cycle TEST PLUS DNA Reagent Kit and Annexin V-FITC Apoptosis Detection Kit I were procured from BD Biosciences Pharmingen (Franklin Lakes, NJ, USA). Mitochondrial Membrane Potential Assay Kit (orange fluorescence) was bought from Abnova (Taipei City, Taiwan). Bax and Bcl-2 Human SimpleStep ELISA® Kits were obtained from Abcam, UK. Caspase Colorimetric Assay Kit was bought from R&D Systems (Minneapolis, MN, USA). All other reagents and chemicals used were of analytical grade and obtained from Sigma-Aldrich (St. Louis, MO, USA).

### 2.2. Plant Materials

The plant (*Manilkara zapota *(L.) P. Royen) was collected from Pahang, Malaysia. The plant with voucher specimen number SK 3179/17 was deposited in Biodiversity Unit of Institute of Bioscience, Universiti Putra Malaysia.

### 2.3. Preparation of Plant Extract

Initially, leaf of* Manilkara zapota *was cut into small pieces and dried in an oven at 40°C for 3 days before being ground into powder form.* Manilkara zapota *leaf sample was extracted with methanol following the method of Tan et al. [13]. About 5 g of samples was extracted with 40 mL of methanol in a shaker (Heidolph Inkubatorhaube, Germany) at 40°C for 2 h. The slurry was filtered using filter paper (Whatman no. 1). The filtrate from the methanol extract was dried using rotary evaporator (Büchi Rotavapor R-200, Switzerland). The yield was measured using electronic balance (Shimadzu, Kyoto, Japan) and stored at -20°C until further analyses. The yield of plant extract was calculated as follows:

(1) Percentage  of  plant  yield  %=Weight  of  plant  extract  (g)Weight  of  plant  sample  (g)×100.

### 2.4. Cell Culture

The human colon carcinoma (HCT-116), human colorectal adenocarcinoma (HT-29), human cervical cancer (HeLa), human hepatocellular carcinoma (HepG2), human gastric adenocarcinoma (HGT-1), human prostate cancer (PC-3), and mouse fibroblast (BALB/c 3T3) cell lines were obtained from American Type Culture Collection (ATCC; Rockville, MD, USA). The HT-29, HCT-116, and HGT-1 cells were grown in DMEM supplemented with 100 IU/mL penicillin, 100 *μ*g/mL streptomycin, and 10% (v/v) FBS. HeLa, HepG2, PC-3, and BALB/c 3T3 cells were cultured in RPMI-1640 medium supplemented with 100 IU/mL penicillin, 100 *μ*g/mL streptomycin, and 10% (v/v) FBS. All cell lines were grown at 5% CO_2_ atmosphere and 37°C humidified atmosphere incubator.

### 2.5. Determination of Cell Viability using 3-(4,5-Dimethylthiazol-2-yl)-2,5-diphenyltetrazolium Bromide (MTT) Assay

The cell viability of HT-29, HCT-116, HeLa, HGT-1, HepG2, PC-3, and BALB/c 3T3 upon treatment with* Manilkara zapota *leaf methanol extract was determined using MTT assay [13]. HT-29, HCT-116, HeLa, HGT-1, HepG2, PC-3, and BALB/c 3T3 cells were seeded at a density of 5 × 10^4^ cells/well in a 96-well plate. After an overnight incubation, the cells were exposed to leaf methanol extract of* Manilkara zapota* (1.56-200 *μ*g/mL). Untreated BALB/c 3T3 and cancer cell lines were included. Following 24, 48, and 72 h of treatment, 20 *μ*L of MTT (5 mg/mL) was added to each well followed by 2-4 h incubation. Lastly, the media from each well were discarded and 100 *μ*L of dimethyl sulfoxide (DMSO) was added to solubilize the purple-blue formazan. The absorbance was read at 570 nm using an ELISA microplate reader (Tecan, Switzerland), and 630 nm was used as a reference wavelength. A graph of percentage of cell viability versus concentration of* Manilkara zapota *leaf methanol extract was plotted, and the concentration of* Manilkara zapota *leaf methanol extract which inhibited 50% of cellular growth as compared to the control (50% inhibitory concentration (IC_50_)) was determined. The cell viability was calculated as follows:

(2) Percentage  of  cell  viability  %=OD570-630  treatmentOD570-630  control×100OD=Optical  density.

### 2.6. Determination of Lactate Dehydrogenase Assay

Cell cytotoxicity was evaluated using an* in vitro *Toxicology Assay Kit by the release of lactate dehydrogenase (LDH), following the manufacturer's protocol. The HCT-116, HT-29, HeLa, HGT-1, HepG2, PC-3, and BALB/c 3T3 cell lines were seeded at a density of 5 × 10^4^ cells/well in a 96-well plate. After 24 h, the cells were exposed to different concentrations of* Manilkara zapota *leaf methanol extract (1.56-200 *μ*g/mL) for 24, 48, and 72 h and, subsequently, the supernatant was collected and used to measure the LDH activity. Untreated BALB/c 3T3 and cancer cell lines were included. The LDH mixtures were added to each well in a volume equal to twice the volume of medium discarded. The reaction was halted after addition of 1/10 (v/v) of 1 N HCl into each well. The absorbance was measured using ELISA microplate reader (Tecan, Switzerland) at a wavelength of 490 nm.

### 2.7. Determination of Cell Morphological Changes

The HeLa cells were seeded at a density of 1 × 10^6^ cells/mL in a 6-well plate. After an overnight incubation, the HeLa cells were exposed to 12, 24, and 48 *μ*g/mL of* Manilkara zapota *leaf methanol extract for 24, 48, and 72 h. The morphological changes and the characteristic of necrosis or apoptosis of the untreated HeLa cells and HeLa cells induced with* Manilkara zapota* leaf methanol extract were viewed under an inverted light microscope (Olympus, Center Valley, PA, USA).

### 2.8. Determination of Cell Cycle Arrest by Flow Cytometer

The cell cycle arrest was measured using CycleTEST PLUS DNA Reagent Kit, following the manufacturer's protocol. The HeLa cells were seeded at a density of 1 × 10^6^ cells in 25 cm^2^ tissue culture flask. After an overnight incubation, the cells were treated with 12, 24, and 48 *μ*g/mL of* Manilkara zapota *leaf methanol extract for 72 h. HeLa cells were then centrifuged at 30 ×* g *for 5 min at room temperature followed by the addition of buffer solution. 250 *μ*L of solution A (trypsin buffer) and 200 *μ*L of solution B (trypsin inhibitor and RNase buffer) were subsequently added to the cells, followed by 10 min incubation at room temperature, respectively. The mixture was then mixed with cold solution C (200 *μ*L of propidium iodide (PI) stain solution) followed by incubation at 4°C for 10 min. The cells were filtered using a 40-*μ*m cell strainer cap. Data acquisition and analysis were measured using NovoCyte Flow Cytometer (ACEA Biosciences, Inc.) with NovoExpress® software.

### 2.9. Determination of Apoptosis by Annexin V-Propidium Iodide Staining

The early and late apoptotic cells activity were evaluated using Annexin V-FITC Apoptosis Detection Kit I, following the manufacturer's instruction. HeLa cells were seeded at a density of 1 × 10^6^ cells in 25 cm^2^ tissue culture flask. After an overnight incubation, the cells were exposed to 12, 24, and 48 *μ*g/mL of* Manilkara zapota *leaf methanol extract for 72 h. After incubation for 72 h, the cells were trypsinized and rinsed twice with phosphate-buffered saline-bovine serum albumin-ethylenediaminetetraacetic acid (PBS-BSA-EDTA) and the cell pellet was resuspended in 100 *μ*L of 1 × binding buffer (0.1 M Hepes/NaOH, pH 7.4 and 1.4 M NaCl_2_, 25 mM CaCl_2_). An aliquot of 10 *μ*L of PI and 5 *μ*L of Annexin V-fluorescein isothiocyanate (FITC) was added to each sample prior to incubation for 10 min in the dark. Lastly, 400 *μ*L of 1 × binding buffer was mixed to the cells and the fluorescence was determined using NovoCyte Flow Cytometer (ACEA Biosciences, Inc.) with NovoExpress software.

### 2.10. Determination of Bax and Bcl-2 Activities in Manilkara Zapota Leaf Methanol Extract

The Bax and Bcl-2 activities were evaluated using Bax and Bcl-2 human SimpleStep ELISA Kits, following the manufacturer's protocol. Briefly, HeLa cells were seeded in 25 cm^2^ tissue culture flask at a density of 1 × 10^5^ cells. After an overnight incubation, the cells were exposed to 12, 24, and 48 *μ*g/mL of* Manilkara zapota *leaf methanol extract for 72 h. The cells were collected and centrifuged at 500 ×* g *at 4°C for 5 min to discard the medium. The cells were rinsed twice with phosphate-buffered saline (PBS) and cold 1× Cell Extraction Buffer PTR and subsequently incubated on ice for 20 min. The cell lysates were then centrifuged at 18,000 ×* g *and 4°C for 20 min, and the supernatants were collected. Bradford protein assay kit was used to quantify the protein concentration. An aliquot of the sample was diluted to desired concentration using 1× Cell Extraction Buffer PTR. About 50 *μ*L of standard or sample was then mixed with 50 *μ*L of antibody cocktail in each well of 96-well plate. The plate was sealed followed by incubation at room temperature for 1 h on a plate shaker set to 400 ×* g*. Each well was rinsed with 3× 350 *μ*L 1× wash buffer PT. An aliquot of 100 *μ*L of TMB substrate was added to each well followed by 10 min incubation in the dark on a plate shaker set to 400 ×* g*. Next, 100 *μ*L of Stop Solution was mixed into each well. The plate was shaken on a plate shaker for 1 min and measured at the wavelength of 450 nm.

### 2.11. Determination of Caspase-3 Assay

The caspase-3 activity was measured spectrophotometrically using a commercial colorimetric assay kit, followed by spectrophotometric detection of the chromophore* p*nitroanilide (*p*NA) after cleavage of the specific substrates DEVD-*p*NA (for caspase-3). Briefly, HeLa cells were seeded at a density of 1 × 10^5^ cells in a 6-well plate. After an overnight incubation, the cells were exposed to 12, 24, and 48 *μ*g/mL of* Manilkara zapota *leaf methanol extract for 72 h. The cells were centrifuged at 250 ×* g *for 10 min to discard the medium. The cell pellets were then lysed in 25 *μ*L of cold lysis buffer, followed by 10 min incubation on ice. The cell lysates were then centrifuged at 10,000 ×* g *and 4°C for 1 min, and the supernatants were collected. Bradford protein assay kit was used to quantify the protein concentration. An aliquot of 50 *μ*L of 2× Reaction Buffer 3 (prior to using the 2× Reaction Buffer 3, 10 *μ*L of DTT was mixed with 1 mL 2× Reaction Buffer 3) was mixed with 50 *μ*L of cell lysate containing 200 *μ*g of total protein, followed by 5 *μ*L of caspase-3 colorimetric substrate (DEVD-*p*Na). Subsequently, the reaction mixture was kept at 37°C for 2 h before being analyzed using ELISA microplate reader (Tecan, Switzerland) at a wavelength of 405 nm.

### 2.12. Determination of Intracellular Reactive Oxygen Species in Manilkara Zapota Leaf Methanol Extract Induces Oxidative Stress

The intracellular reactive oxygen species (ROS) in HeLa cells treated with* Manilkara zapota *leaf methanol extract was measured using dichlorodihydrofluorescein diacetate (DCFH-DA). Initially, HeLa cells were seeded at a density of 1 × 10^5^ cells/well in a 6-well plate overnight and preincubated with 10 *μ*M DCFH-DA in complete media for 1 h. DCFH-DA was discarded and washed twice with PBS, followed by treatment with* Manilkara zapota *leaf methanol extract (12, 24, and 48 *μ*g/mL) for 72 h. Following 72 h of incubation, all adherent and floating cells were collected. The sample was analyzed using NovoCyte Flow Cytometer (ACEA Biosciences, Inc.) with NovoExpress software.

### 2.13. Determination of Mitochondrial Membrane Potential

The Mitochondrial Membrane Potential Assay Kit (orange fluorescence) (Abnova, Taipei City, Taiwan) was used to measure the alteration of mitochondrial membrane potential (MMP). Initially, HeLa cells were seeded at a density of 1 × 10^5^ cells/well in a 25 cm^2^ tissue culture flask. After an overnight incubation, the cells were exposed to 12, 24, and 48 *μ*g/mL of* Manilkara zapota *leaf methanol extract for 72 h. After treatment, the cells were trypsinized, rinsed twice in PBS, and the cells were suspended in 1 mL of Assay Buffer. After adding 2 *μ*L of 500× MitoLite Orange, the cells were incubated at 37°C and 5% CO_2_ incubator for 30 min. The cells were centrifuged at 500 ×* g* for 4 min. Lastly, the cells were resuspended in 1 mL of Assay Buffer. The fluorescence intensity was measured using NovoCyte Flow Cytometer (ACEA Biosciences, Inc.) with NovoExpress software.

### 2.14. Determination of Catalase Activity

Initially, HeLa cells were seeded at a density of 1 × 10^5^ cells for 24 h. The cells were then treated with 12, 24, and 48 *μ*g/mL of* Manilkara zapota *leaf methanol extract for 72 h. HeLa cells were centrifuged at 250 ×* g *for 10 min to discard the supernatant. The cell pellets were subsequently lysed in 100 *μ*L of cold lysis buffer prior to being incubated for 10 min on ice. Following centrifugation at 10,000 ×* g *and 4°C for 1 min, the supernatants were collected and kept at -80°C for catalase assay. Catalase level was determined using the method described by Aebi [14]. A 1.9-mL aliquot of the phosphate buffer (0.05 M, pH 7.0) was mixed with 0.1 mL of supernatant and 1 mL of hydrogen peroxide (0.019 M). The absorbance was measured at a wavelength of 240 nm using UV-visible spectrophotometer (Pharmaspec UV-1700, Shimadzu, Kyoto, Japan). The catalase activity was expressed as nmol H_2_O_2_ consumed min^−1^ mg^−1^ protein.

### 2.15. Total RNA Extraction and cDNA Synthesis

Total ribonucleic acid (RNA) was isolated using TRI Reagent®, following the manufacturer's instruction. The HeLa cells were seeded at a density of 1 × 10^5^ cells in a 25 cm^2^ culture flask for 24 h. After being treated with 12, 24, and 48 *μ*g/mL of* Manilkara zapota *leaf methanol extract for 72 h, the cells were homogenized and the cell lysates were aliquot in falcon tubes. An aliquot of 1 mL TRI Reagent was added in falcon tubes and resuspended. About 100 *μ*L of 1-bromo-3-chloropropane per mL of TRI Reagent used was added and vortexed vigorously for 15 s prior to incubation for 2–15 min at room temperature. After centrifugation for 15,000 × g and 2-8°C for 15 min, the mixture was divided into a lower red organic layer, an interphase, and a colorless upper aqueous layer containing RNA. The aqueous layer was precipitated after the addition of 500 *μ*L of isopropanol. The sample was kept for 5-10 min at room temperature before being centrifuged at 11,500 ×* g* and 2-8°C for 10 min. The supernatant was discarded and the RNA pellet was rinsed with 1 mL of 75% (v/v) ethanol followed by centrifugation at 5,500 ×* g *and 2-8°C for 5 min. An aliquot of 50 *μ*L of RNase-free water was mixed with RNA pellet and resuspended before being stored at -80°C. About 2 *μ*g of total RNA per 20 *μ*L was reverse-transcribed using High Capacity RNA-to-cDNA Kit, following the manufacturer's instructions. The reverse transcription reaction was conducted using an Authorized Thermal Cycler.

### 2.16. Quantitative Real-Time Polymerase Chain Reaction Analysis

Quantitative real-time PCR was performed using SYBR® Select Master Mix (CFX).  Table 1 shows the nucleotide primer sequences originating from human cell lines. The specific primers were validated for amplification efficiency over a concentration range and consistency with the amplification efficiency of housekeeping genes and amplification specificity. The mRNA levels of* cytochrome c*, epidermal growth factor receptor (*EGFR*), and nuclear factor-kappa B (NF-*κ*B) were assayed using SYBR Select Master Mix, CFX in a final volume of 20 *μ*L, following the manufacturer's instructions. Briefly, the primers, kit contents (RNase-free water and SYBR Select Master Mix (CFX)), and cDNA template were thawed on ice. The qPCR reaction was determined based on the following conditions: (1) 50°C for 120 s (1 cycle) for uracil-DNA glycosylase (UDG) activation; (2) 95°C for 120 s (1 cycle) for DNA polymerase activation; (3) 95°C for 2 s (40 cycles) for denaturation; and (4) 60°C for 30 s (40 cycles) for annealing/extension. All the controls and samples were evaluated in triplicate using the BioRAD-iQ™ 5 Multicolor Real-Time PCR Detection System (Hercules, CA, USA), and CFX Manager™ software (version 1.6, Bio-Rad, Hercules, CA, USA) was used for data analysis. The housekeeping genes (18S rRNA, glyceraldehyde-3-phosphate dehydrogenase (*GAPDH*), and beta-actin (*ACTB*)) were used for normalization.

### 2.17. Determination of Total Phenolic Content

Total phenolic content (TPC) in plant extract was measured by using Folin-Ciocalteu's reagent following a modified method by Meda et al. [15]. An aliquot of 0.3 mL of plant extract was mixed with 1.2 mL of sodium carbonate (7.5% (w/v)) and 1.5 mL of Folin-Ciocalteu's reagent (diluted 10 times). The mixture was vortexed prior to being incubated at room temperature for 30 min. The absorbance of the sample was read at 765 nm using a UV–visible spectrophotometer (Pharmaspec UV-1700, Shimadzu, Kyoto, Japan). The TPC value of the sample was expressed in milligram gallic acid equivalents per gram of extract (mg GAE/g extract).

### 2.18. Determination of Total Flavonoid Content

Total flavonoid content (TFC) in plant extract was measured following a modified method by Shanmugapriya et al. [16]. An aliquot of 0.5 mL of plant extract solution was added to 0.1 mL of 10% aluminium chloride hexahydrate and 1.5 mL of 95% ethanol. The mixture was then added to 2.8 mL distilled water and 0.1 mL of potassium acetate (1 M). The absorbance of the sample was read at 415 nm after 40 min incubation at room temperature. An equal volume (0.1 mL) of distilled water was substituted with 10% aluminium chloride hexahydrate as the blank. The total flavonoid content was expressed in milligram quercetin equivalents per 100 gram of extract (mg QE/100 g).

### 2.19. Beta-Carotene Bleaching Test

The *β*-carotene bleaching assay was performed as previously described by Tan et al. [13]. An aliquot of 5 mg of *β*-carotene was added to 10 mL of chloroform, and 3 mL of this mixture was transferred to 100 mL round-bottom flask. The chloroform was evaporated at 40°C by vacuum evaporation. After evaporation, about 40 mg of linoleic acid and 400 mg of Tween 40 emulsifier were mixed with 100 mL of distilled water, followed by vigorous shaking. A 4.8-mL aliquot of this emulsion was mixed with extract or 200 *μ*L of methanol (control). The standard antioxidant used was butylated hydroxytoluene (BHT). After the addition of this emulsion into a series of test tubes, the zero time absorbance was read at a wavelength of 470 nm using UV-visible spectrophotometer (Pharmaspec UV-1700, Shimadzu, Kyoto, Japan). The subsequent absorbance was read over 2 h periods at every 20 min followed by incubation of the test tubes at 50°C. The blank samples were used for background subtraction. The capacity of the plant extract to protect against *β*-carotene oxidation was calculated as follows:

(3) At-0 Sample−At-0 Blank–At-120 min Sample−At-120 min BlankAt-0 Control−At-120 min Control=CBeta-carotene  retention  %=100%−C×100%A=absorbance  at  a  particular  time,  C=carotene  depletion  factor.

### 2.20. Determination of 1, 1-Diphenyl-2-picryl-hydrazyl (DPPH) Radical Scavenging Capacity

The 1,1-diphenyl-2-picryl-hydrazyl (DPPH) radical scavenging capacity was evaluated by UV-visible spectrophotometer (Pharmaspec UV-1700, Shimadzu, Kyoto, Japan) [17]. A 1.5-mL aliquot of DPPH (0.1 nM) in methanol was mixed with 0.5 mL sample. The solution was vortexed for 15 s prior to being incubated at room temperature for 1 h. The absorbance of the sample was read at a wavelength of 517 nm. Control (without sample) and standard (ascorbic acid) were prepared using the same methodology. DPPH assay is expressed as effective concentration (EC_50_), the concentration which is needed to scavenge 50% of the DPPH free radicals.

### 2.21. Determination of Polyphenols using Ultra Performance Liquid Chromatography (UPLC)

Polyphenols quantification in plant extract was carried out using Agilent Technologies 1290 Infinity model G4220A equipped with a diode array detector setup wavelength of 280 nm and 320 nm. Chromatographic separation was analyzed using a LiChroCART® 250-4, 6 C18 column (5 *μ*m, 250 mm × 4.6 mm). Solvent (A) water-acetic acid (94:6, v/v, pH 2.27) and solvent (B) acetonitrile were used as the mobile phase. These gradient elution condition and solvent composition have been described earlier by Tan et al. [18]. The solvent gradients were as follows: 0-15% B for 40 min, 15-45% B for 40 min, and 45-100% B for 10 min with a flow rate of 0.5 mL/min. An aliquot of 20 *μ*L sample was injected. The mobile phase and sample were filtered using a Millipore filter of 0.22 *μ*m. The polyphenolic compounds were quantified by comparing their retention times with the calibration curves of their respective standards (caffeic acid, syringic acid, vanillic acid, ferulic acid, gallic acid, and p-coumaric acid).

### 2.22. Qualitative Analysis of Phytochemicals

The leaf methanol extract of* Manilkara zapota *was analyzed for the presence of flavonoids, steroids, saponins, phlobatannins, and triterpenoids following the methods as previously reported by Harborne [19] and Evans [20].

#### 2.22.1. Qualitative Analysis of Steroids

Steroids in* Manilkara zapota *leaf methanol extract were determined using Salkwoski's test. About 0.5 g of the plant extract was mixed with 2 mL of chloroform. The sulphuric acid was added to the mixture to form a layer. A reddish brown color formed at the interface which shows the presence of steroids.

#### 2.22.2. Qualitative Analysis of Triterpenoids

Triterpenoids in* Manilkara zapota *leaf methanol extract were evaluated using Hishorn's test. About 0.5 g of the plant extract was added to 2 mL of chloroform. The mixture was then mixed with 2 mL of trichloroacetic acid (TCA) followed by incubation for 10 min. The changes from yellow to red color indicate the presence of triterpenoids.

#### 2.22.3. Qualitative Analysis of Flavonoids

Flavonoids were determined using ferric chloride test. About 0.5 g of the* Manilkara zapota *leaf methanol extract was boiled in distilled water prior to being filtered. Two or three drops of 10% ferric chloride were subsequently mixed with 2 mL of the filtrate. A green-blue or violet color shows the presence of flavonoids.

#### 2.22.4. Qualitative Analysis of Saponins

Saponins in* Manilkara zapota *leaf methanol extract were evaluated using frothing test. About 1 g of the plant extract was mixed with 3 mL of distilled water and vortexed vigorously for 5 min. The presence of frothing indicates the presence of saponins.

#### 2.22.5. Qualitative Analysis of Phlobatannins

The presence of a red precipitate after the plant extract boiled with 1% hydrochloric acid indicates the presence of phlobatannins.

### 2.23. Statistical Analysis

The data are presented as the mean ± standard deviation (SD) using one-way analysis of variance (ANOVA). The differences with* P* < 0.05 were considered significant. The statistical analyses were carried out using the Statistical Package for Social Science (SPSS) version 19.0.

## 3. Results and Discussion

### 3.1. The Yield of Manilkara Zapota Leaf Methanol Extract

Extraction yield does depend on the extraction method but also on the extraction solvent. Polar solvents are commonly used for recovering polyphenols from plant matrices. Methanol has been reported to be more efficient in the extraction of low molecular weight polyphenols [21]. It can be seen that the extraction yield of pure methanol (31.06 ± 1.54%) was significantly higher than that of 70% ethanol (8.37 ± 0.40%) and water (8.76 ± 1.46%) (*P* < 0.05) (unpublished data). This result indicates that compounds other than phenolic may have been extracted and thus contribute to the high yield.

### 3.2. Manilkara Zapota Leaf Methanol Extract Decreases Viability of HeLa Cells

**Figure 1:**
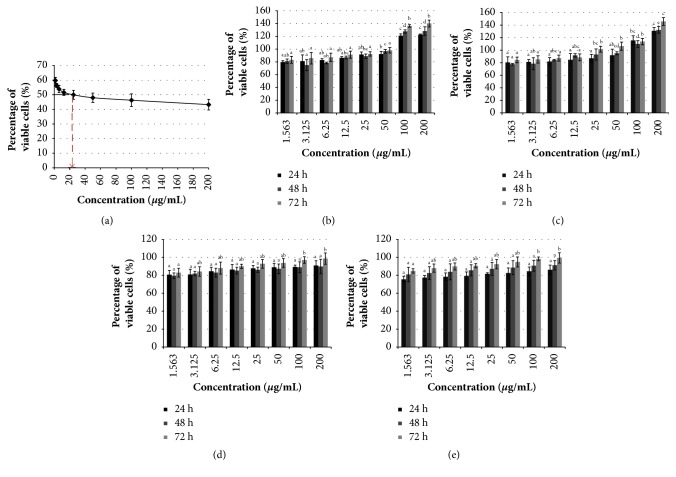
Treatment of* Manilkara zapota *leaf methanol extract on cancer cells. (a) Treatment of* Manilkara zapota *leaf methanol extract on HeLa cells. The cell viability was evaluated by 3-(4,5-dimethylthiazol-2-yl)-2,5-diphenyltetrazolium bromide (MTT) assay after 72 h exposure with* Manilkara zapota *leaf methanol extract. (b)* Manilkara zapota *leaf methanol extract increases proliferation of human prostate cancer (PC-3) cells after 24, 48, and 72 h using MTT assay. (c)* Manilkara zapota *leaf methanol extract promotes proliferation of PC-3 cells after 24, 48, and 72 h evaluated by lactate dehydrogenase (LDH) assay. (d) Treatment of* Manilkara zapota *leaf methanol extract in mouse fibroblast (BALB/c 3T3) cell lines evaluated using MTT assay. (e) Cell viability of BALB/c 3T3 cell lines after treatment with* Manilkara zapota *leaf methanol extract was evaluated using LDH assay. Values are reported as mean ± SD (n = 3). Value with different superscript letter indicates significant difference between groups by Tukey's test (*P* < 0.05).

To determine the antiproliferative effect of* Manilkara zapota* leaf methanol extract on cancer cells, human colon carcinoma (HCT-116), human colorectal adenocarcinoma (HT-29), human cervical cancer (HeLa), human gastric adenocarcinoma (HGT-1), human hepatocellular carcinoma (HepG2), human prostate cancer (PC-3), and mouse fibroblast (BALB/c 3T3) cell lines were exposed to different concentrations of* Manilkara zapota *leaf methanol extract (1.56-200 *μ*g/mL) for 24, 48, and 72 h, and the effects on cell viability were evaluated using 3-(4,5-dimethylthiazol-2-yl)-2,5-diphenyltetrazolium bromide (MTT) assay. We found that* Manilkara zapota* leaf methanol extract was cytotoxic to all cancer cells studied after 72 h incubation (Table 2). According to published guidelines, any extract that possesses potentially cytotoxic activity should have an IC_50_ less than 100 *μ*g/mL [22]. As shown in Table 2,* Manilkara zapota* leaf methanol extract inhibited the growth of HT-29 cells after 24, 48, and 72 h, with IC_50 _value 93.27 ± 17.19, 89.29 ± 6.01, and 69.12 ± 8.10 *μ*g/mL, respectively. Consistent with the cytotoxic effect observed in HT-29 cells,* Manilkara zapota* leaf methanol extract also decreases the viability of HCT-116 cells in a time-dependent manner after 24 h (90.14 ± 14.23 *μ*g/mL), 48 h (87.33 ± 9.29 *μ*g/mL), and 72 h (83.17 ± 9.92 *μ*g/mL). A similar trend was also observed in HGT-1 and HepG2 cells. We found that, after treatment with* Manilkara zapota *leaf methanol extract for 72 h, both HGT-1 (49.44 ± 10.62 *μ*g/mL) and HepG2 (73.02 ± 9.33 *μ*g/mL) cells were inhibited. HeLa cells were relatively more sensitive to* Manilkara zapota* leaf methanol extract than other cancer cell lines studied. It suppressed the viability of HeLa cells in a time-dependent manner, with IC_50_ values 89.29 ± 18.20, 59.23 ± 10.33, and 23.87 ± 5.02 *μ*g/mL for 24, 48, and 72 h, respectively.  Figure 1(a) shows the percentage of viable HeLa cells after 72 h exposure to* Manilkara zapota* leaf methanol extract. Conversely, we observed that* Manilkara zapota* leaf methanol extract promotes proliferation of PC-3 cells after 24, 48, and 72 h incubation (Figure 1(b)). Thus, we believe that PC-3 cells were relatively more resistant to this extract compared to other cancer cell lines. However, the molecular mechanisms underlying PC-3 cells in this extract warrants further elucidation.

To verify the cytotoxicity activity of* Manilkara zapota* leaf methanol extract, the proliferation of all cancer cells studied was evaluated using lactate dehydrogenase (LDH) assay. Cells treated with different concentrations of* Manilkara zapota *leaf methanol extract (1.56-200 *μ*g/mL) were harvested and subjected to LDH analysis. Consistent with MTT results, LDH analyses demonstrated that both cells viabilities of HCT-116 and HT-29 were reduced after treatment with* Manilkara zapota *leaf methanol extract (Table 2). Compared to other cancer cell lines studied, HeLa cells is the most sensitive towards* Manilkara zapota *leaf methanol extract with an IC_50_ value 25.76 ± 8.93 *μ*g/mL after 72 h incubation. Conversely, HGT-1 and HepG2 cells were less sensitive compared to HeLa cells (Table 2). Consistent with the cytotoxic effect observed in MTT assay, our LDH analysis further demonstrated that* Manilkara zapota *leaf methanol extract promotes proliferation of PC-3 cells after 24, 48, and 72 h (Figure 1(c)). Interestingly, no cytotoxicity was observed in* Manilkara zapota *leaf methanol extract in BALB/c 3T3 cell lines as evaluated using both MTT and LDH assays (Figures 1(d) and 1(e)). Taken together, our data suggest that* Manilkara zapota *leaf methanol extract can induce cytotoxicity in different cancer cell lines, in which HeLa cells are being the most sensitive compared to other cancer cells studied. Thus, HeLa cells were selected for further analyses. Given the wide cytotoxicity range of* Manilkara zapota *leaf methanol extract against HeLa cells as evaluated using MTT and LDH assays, only these three concentrations (12, 24, and 48 *μ*g/mL) were selected for further analyses.

### 3.3. Manilkara Zapota Leaf Methanol Extract Induces Morphological Changes of HeLa Cells

**Figure 2:**
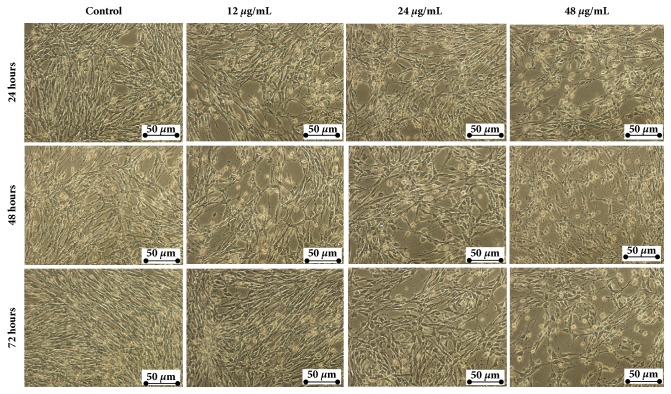
*Manilkara zapota *leaf methanol extract induces morphological changes and decreases the proliferation of human cervical cancer (HeLa) cells. HeLa cells were incubated with 12, 24, and 48 *μ*g/mL of* Manilkara zapota *leaf methanol extract for 24, 48, and 72 h and then observed under an inverted light microscope (Magnification 200×).

**Figure 3:**
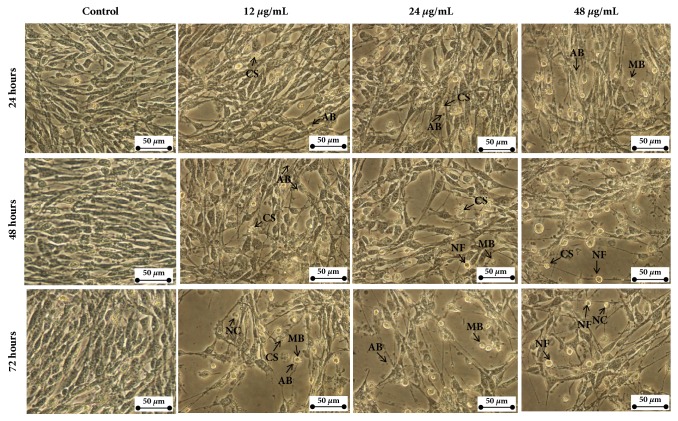
Close-up view of morphological changes in HeLa cells after treatment with* Manilkara zapota *leaf methanol extract at 12, 24, and 48 *μ*g/mL of* Manilkara zapota *leaf methanol extract for 24, 48, and 72 h and observed under an inverted light microscope (Magnification 400×). The cells showed the typical characteristics of apoptosis such as cellular shrinkage (CS), apoptotic bodies (AB), nuclear fragmentation (NF), nuclear compaction (NC), and membrane blebbing (MB).

To explore the morphological changes of HeLa cells treated with* Manilkara zapota *leaf methanol extract, HeLa cells were exposed to different concentrations of the extract (12, 24, and 48 *μ*g/mL). As depicted in Figure 2, increasing concentration of* Manilkara zapota *leaf methanol extract from 12 to 48 *μ*g/mL for 24, 48, and 72 h incubation led to cell morphological changes and decrease in the number of cells (Figure 2). The proliferation of cells treated with 48 *μ*g/mL of* Manilkara zapota *leaf methanol extract for 24 h and 48 h was inhibited and this phenomenon became obvious at 72 h (Figure 2). The marked detachment was observed in HeLa cells exposed to 12 *μ*g/mL and in the latter (24 and 48 *μ*g/mL) from 24 h to 72 h (Figure 3). Additionally, we also observed cell rounding accompanied with a typical apoptotic morphology including chromatin condensation (CC), membrane blebbing (MB), apoptotic bodies (AB), nuclear fragmentation (NF), and nuclear compaction (NC) (Figure 3). Based on the findings, leaf methanol extract of* Manilkara zapota* induced cells inhibition and showed the obvious typical characteristic of apoptotic cells after 72 h incubation. Therefore, an incubation time of 72 h was selected for further analyses.

### 3.4. Manilkara Zapota Leaf Methanol Extract Induces Cell Cycle Arrest in HeLa Cells

**Figure 4:**
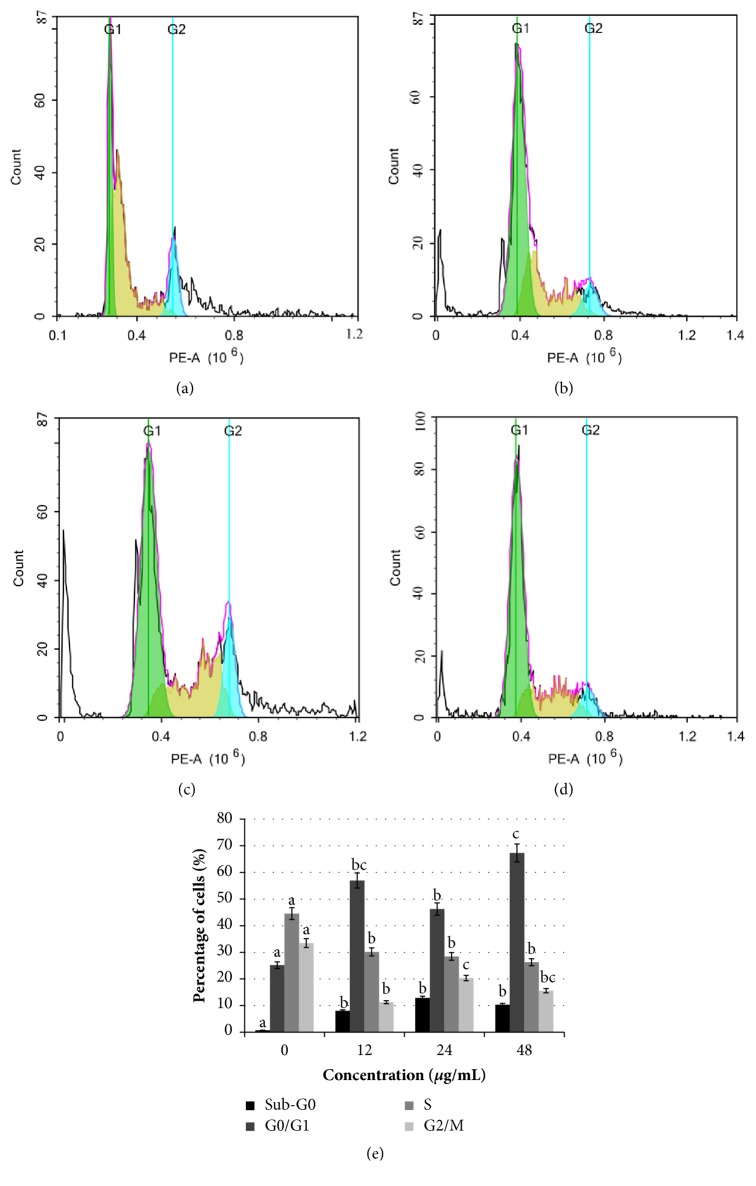
Assessment of cell cycle kinetics in (a) untreated HeLa cells and HeLa cells treated with* Manilkara zapota *leaf methanol extract at concentrations of (b) 12 *μ*g/mL, (c) 24 *μ*g/mL, and (d) 48 *μ*g/mL for 72 h, and the cell cycle kinetic was determined by flow cytometry. (e) The cell cycle analysis was determined using propidium iodide (PI) staining and analyzed by flow cytometry. Values are reported as mean ± SD (n = 3). Value with different superscript letter indicates significant difference between groups by Tukey's test (*P* < 0.05).

To examine if the cytotoxic activity of* Manilkara zapota* leaf methanol extract was due to the cell cycle arrest and induction of apoptosis, HeLa cells were exposed to different concentrations of* Manilkara zapota* leaf methanol extract (12, 24, and 48 *μ*g/mL) for 72 h, the cell apoptosis was evaluated by detecting the sub-G_0_ population upon propidium iodide (PI) staining and analyzed by flow cytometry. As illustrated in Figure 4(e), exponentially growing of untreated HeLa cells contained a low level (0.75%) of apoptotic cells, which is significant difference between the untreated cells and those from the groups treated with 12 *μ*g/mL, 24 *μ*g/mL, or 48 *μ*g/mL of* Manilkara zapota *leaf methanol extract (*P* < 0.05). This finding indicates that* Manilkara zapota *leaf methanol extract induces population sub-G_0_ phase following treatment with* Manilkara zapota *leaf methanol extract (Figure 4(e)), indicate DNA degradation due to the activation of endogenous nucleases during apoptosis [23]. Treatment with* Manilkara zapota *leaf methanol extract for 72 h significantly increased the percentage of cells at G_0_/G_1_ phase as compared to the untreated cells (*P* < 0.05) with a concomitant decrease of the S phase at 72 h (Figure 4(e)). This result implies that* Manilkara zapota *leaf methanol extract regulates several biological processes associated with cell survival and death. Our findings presented in this study demonstrated that* Manilkara zapota *leaf methanol extract destroys HeLa cells in dividing state. Overall, both cell viability and flow cytometric assays suggest that* Manilkara zapota *leaf methanol extract can indeed result in cytotoxicity.

### 3.5. Manilkara Zapota Leaf Methanol Extract Induces Apoptosis in HeLa Cells

**Figure 5:**
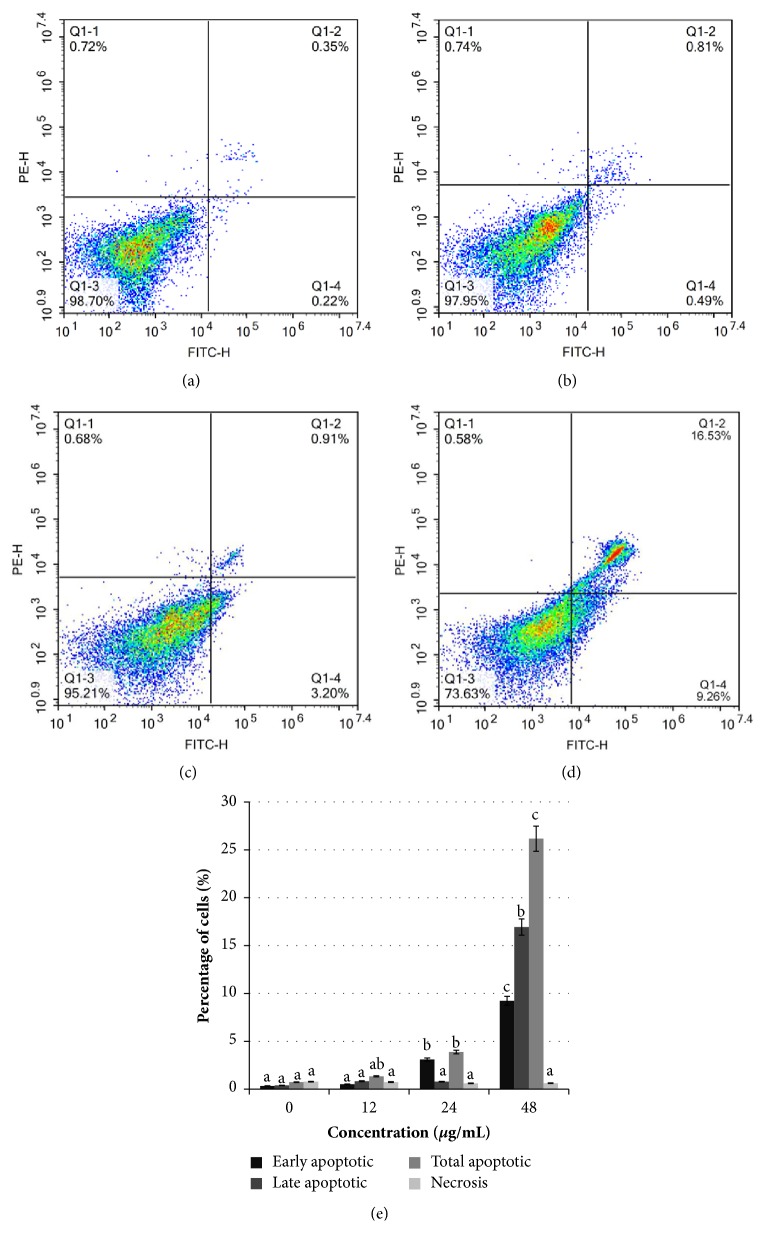
Evaluation of* Manilkara zapota *leaf methanol extract-induced apoptotic cell death in (a) untreated HeLa cells and HeLa cells treated with (b) 12 *μ*g/mL, (c) 24 *μ*g/mL, and (d) 48 *μ*g/mL of* Manilkara zapota *leaf methanol extract for 72 h. (e) Assessment of apoptotic cell death treated with* Manilkara zapota *leaf methanol extract was determined using Annexin V-FITC and propidium iodide (PI) staining assay using flow cytometry. Values are reported as mean ± SD (n = 3). Value with different superscript letter indicates significant difference between groups by Tukey's test (*P* < 0.05).

To further confirm* Manilkara zapota* leaf methanol extract induces apoptosis in HeLa cells, Annexin V-FITC/PI double-staining followed by flow cytometry was conducted in HeLa cells upon exposure to* Manilkara zapota* leaf methanol extract (12, 24, and 48 *μ*g/mL) for 72 h. We observed that treatment with 24 and 48 *μ*g/mL of* Manilkara zapota *leaf methanol extract led to a significant increase in the percentage of early apoptotic cells compared to the control (*P* < 0.05) (Figure 5(e)). The late apoptotic cells in HeLa cells treated with 48 *μ*g/mL* Manilkara zapota *leaf methanol extract for 72 h were significantly increased compared to the control (*P* < 0.05). Overall, treatment with 24 and 48 *μ*g/mL* Manilkara zapota *leaf methanol extract for 72 h significantly increased the total apoptotic HeLa cells compared to the control (*P* < 0.05), with a maximum effect noted at a concentration of 48 *μ*g/mL. Our results suggest that* Manilkara zapota *leaf methanol extract induces translocation of phosphatidylserine from inner to the outer leaflet of the cell membrane, which indicates a hallmark of apoptosis. Importantly, we found that the percentage of total apoptotic cells was more prominent than necrotic cells (<1%). This finding implied that* Manilkara zapota *leaf methanol extract might be used as a therapeutic agent for human cervical cancer. Taken together, our data demonstrate that* Manilkara zapota *leaf methanol extract potentiates the apoptotic effects on HeLa cells rather than necrosis.

### 3.6. Manilkara Zapota Leaf Methanol Extract Modulates Bcl-2 Family in HeLa Cells

**Figure 6:**
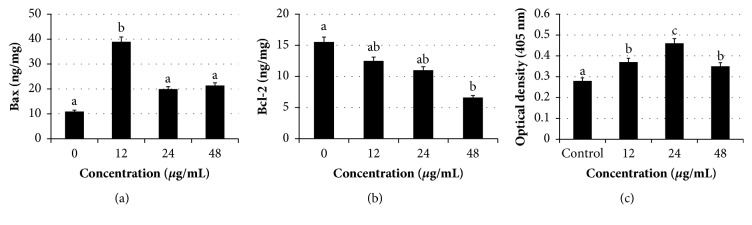
Apoptotic activities of* Manilkara zapota *leaf methanol extract on HeLa cells after 72 h incubation. HeLa cells treated with 12 and 48 *μ*g/mL of* Manilkara zapota *leaf methanol extract upregulated apoptotic protein expression of (a) Bax and downregulated (b) Bcl-2, respectively. (c)* Manilkara zapota *leaf methanol extract-induced caspase-3 activation. Values are reported as mean ± SD (n = 3). Value with different superscript letter indicates significant difference between groups by Tukey's test (*P* < 0.05).

To examine whether apoptosis induction of* Manilkara zapota* leaf methanol extract in HeLa cells involved the proapoptotic protein (Bax) and antiapoptotic protein expression (Bcl-2), the Bax and Bcl-2 protein expression in HeLa cells following treatment with 12, 24, and 48 *μ*g/mL* Manilkara zapota* leaf methanol extract was evaluated (Figures 6(a) and 6(b)). Extensive research has shown that many cellular organelles such as endoplasmic reticulum, mitochondria, lysosomes, and Golgi apparatus play a critical role in apoptotic cell death [24, 25]. The mitochondria have a particularly prominent role in apoptosis and Bcl-2 family proteins are central players in mitochondria-mediated cell death and survival [26]. Phosphorylation of Bcl-2 may be required to trigger its antiapoptotic functions [27]. In the present study, the cells treated with 12 *μ*g/mL of* Manilkara zapota *leaf methanol extract significantly increased the proapoptotic role of Bax compared to the untreated cells (*P *< 0.05). In addition, our data also revealed that 48 *μ*g/mL* Manilkara zapota *leaf methanol extract induced the phosphorylation of Bcl-2, suggesting that the Bcl-2 phosphorylation (Figure 6(b)) and Bax activation (Figure 6(a)) may correlate with apoptotic cells death. Taken together, this finding indicates a crucial role of the Bcl-2 protein in* Manilkara zapota* leaf methanol extract-induced apoptotic cell death.

### 3.7. Manilkara Zapota Leaf Methanol Extract Activates Caspase-Dependent Apoptotic Pathway

To verify whether the growth suppressive activity could be dependent on the stimulation of caspase-3 activity, which plays a crucial role in the regulation of apoptotic responses [28], the intracellular levels of caspase-3 in HeLa cells after exposure to* Manilkara zapota* leaf methanol extract (12, 24, and 48 *μ*g/mL) were evaluated. As shown in Figure 6(c), treatment with* Manilkara zapota* leaf methanol extract showed apparently upregulation in caspase-3 activity compared to the control (*P* < 0.05). Indeed, quantification of caspase-3 enzymatic activity confirmed the caspase activation by leaf methanol extract of* Manilkara zapota*. Collectively, these findings indicated that* Manilkara zapota* leaf methanol extract induced apoptosis involved caspase-dependent pathway in HeLa cell.

### 3.8. Manilkara Zapota Leaf Methanol Extract Triggers Apoptosis via ROS-Mediated Mitochondrial Pathway

**Figure 7:**
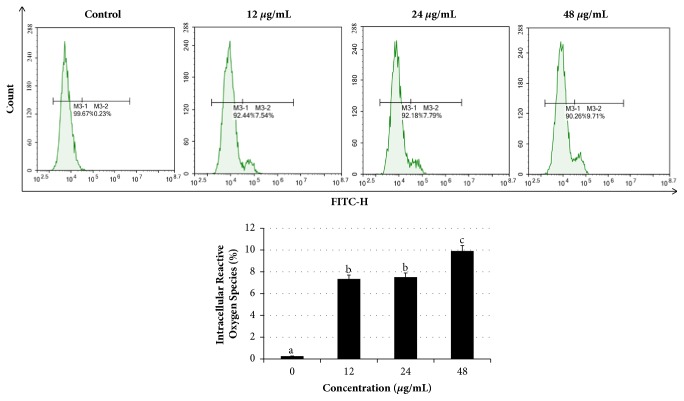
*Manilkara zapota *leaf methanol extract induces cell apoptosis involved in reactive oxygen species (ROS) production in HeLa cells. The levels of ROS were determined with DCFH-DA staining by flow cytometry after 72 h treatment with* Manilkara zapota *leaf methanol extract. Values are reported as mean ± SD (n = 3). Value with different superscript letter indicates significant difference between groups by Tukey's test (*P* < 0.05).

To investigate whether the inhibitory effects of* Manilkara zapota* leaf methanol extract in HeLa cells involved oxidative stress, we evaluated using reactive oxygen species (ROS) sensitive dye dichlorodihydrofluorescein diacetate (DCFH-DA). Our data presented in this study showed that after exposure to 12, 24, and 48 *μ*g/mL of* Manilkara zapota* leaf methanol extract significantly increased ROS generation as compared to the untreated cells (control) (*P* < 0.05) (Figure 7). This finding indicates that induction of intracellular ROS in HeLa cells which might be responsible for induction of apoptosis is due to the oxidative stress induced by* Manilkara zapota *leaf methanol extract.

**Figure 8:**
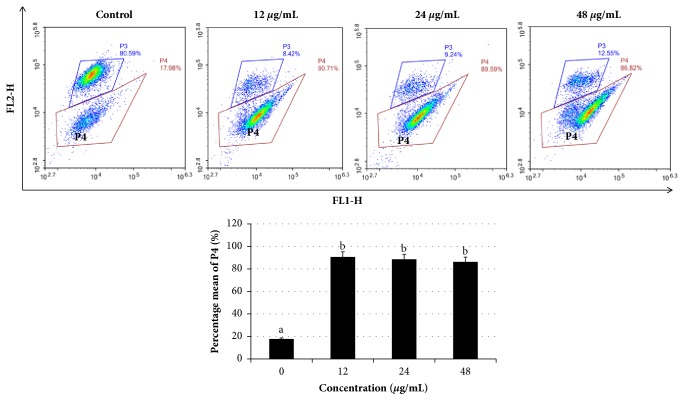
*Manilkara zapota *leaf methanol extract induced loss of mitochondria membrane potential. HeLa cells were incubated with 12, 24, and 48 *μ*g/mL of* Manilkara zapota *leaf methanol extract for 72 h and staining with MitoLite Orange. The fluorescence intensity was measured using NovoCyte Flow Cytometer with NovoExpress software. Values are reported as mean ± SD (n = 3). Value with different superscript letter indicates significant difference between groups by Tukey's test (*P* < 0.05).

Loss of mitochondrial membrane potential is an early event during apoptosis. Because excessive ROS accumulation may contribute oxidative stress and mitochondrial dysfunction, we evaluated mitochondrial function using MitoLite Orange, an indicator of mitochondrial membrane potential, by flow cytometry analysis. Our data revealed that mitochondrial membrane potential in cells treated with* Manilkara zapota *leaf methanol extract was significantly decreased compared with the control (*P* < 0.05) (Figure 8). These data indicate that* Manilkara zapota *leaf methanol extract induces depolarization and mitochondrial membrane potential collapse in cells leading to activation of apoptosis. As we know, chemotherapy agents increase oxidative stress and result in ROS accumulation [29]. The generation of ROS in the mitochondria could suppress the mitochondrial respiration chain, which causes mitochondrial membrane rupture and apoptotic cell death [30]. Collectively, the data presented in this study suggest that* Manilkara zapota *leaf methanol extract may modulate apoptosis through the ROS-mediated mitochondrial pathway.

### 3.9. Manilkara Zapota Leaf Methanol Extract Promotes Catalase Activity

To test whether the apoptotic effects of* Manilkara zapota* leaf methanol extract in HeLa cells may be associated with the antioxidant enzyme, we evaluated the catalase activity. The decrease of catalase levels in the untreated cells (control) (6.94 ± 0.01 nmol H_2_O_2_ consumed min^−1^ mg^−1^ protein) demonstrated that the defense mechanism may have been overwhelmed to ameliorate the amount of hydrogen peroxide ions generated on the surface of the cells. The observed effect may also be due to the impairment of the antioxidant enzyme, which serves as a safeguard for cells during ROS detoxification [31]. This result indicates that untreated cells exhibited a reduction of catalase level associated with a reduction of antioxidative capacity. Conversely, the catalase levels in the treatment groups [12 *μ*g/mL (8.57 ± 0.03 nmol H_2_O_2_ consumed min^−1^ mg^−1^ protein), 24 *μ*g/mL (10.29 ± 0.01 nmol H_2_O_2_ consumed min^−1^ mg^−1^ protein), and 48 *μ*g/mL of* Manilkara zapota *leaf methanol extract (9.08 ± 0.04 nmol H_2_O_2_ consumed min^−1^ mg^−1^ protein)] were significantly increased compared with that of the control group (6.94 ± 0.01 nmol H_2_O_2_ consumed min^−1^ mg^−1^ protein) (*P* < 0.05).

Numerous anticancer agents induce apoptotic cell death via induction of oxidative stress to a threshold that compromises the cell proliferation, thus resulting in an imbalance between antioxidant and ROS within cancer cells [32]. Our present study found that exposure to* Manilkara zapota *leaf methanol extract increased catalase activity. It is proposed that the antioxidant defense system in HeLa cells is activated in response to the accumulation of cellular oxidative stress produced by* Manilkara zapota *leaf methanol extract. Catalase is a vital endogenous antioxidant enzyme that detoxifies hydrogen peroxide to water and oxygen, thereby limiting the adverse effects of ROS [33]. Interestingly, plants commonly exerted antioxidant activity, in which some of them are demonstrated to have distinguished apoptosis-inducing ability via induction of oxidative stress [34, 35]. In this context, catalase was elevated in order to scavenge the ROS generation induced by* Manilkara zapota *leaf methanol extract. Collectively, our results suggest that ROS level induced by the extract was high and has surpassed the antioxidant capacity, thus resulting in apoptosis in HeLa cells.

### 3.10. Manilkara Zapota Leaf Methanol Extract Triggers Release of Cytochrome c

**Figure 9:**
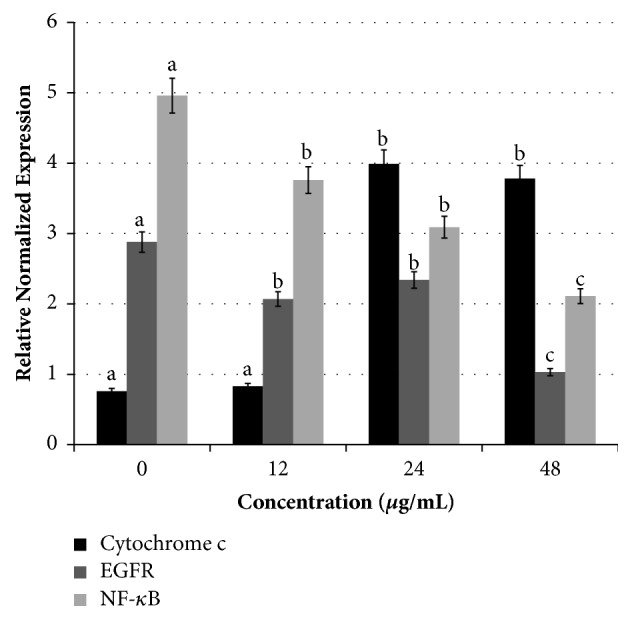
mRNA levels of* cytochrome c*, nuclear factor-kappa B (NF-*κ*B), and epidermal growth factor receptor (*EGFR*) in HeLa cells treated with* Manilkara zapota *leaf methanol extract for 72 h and evaluated using quantitative real-time polymerase chain reaction (PCR). Values are reported as mean ± SD (n = 3). Value with different superscript letter indicates significant difference between groups by Tukey's test (*P *< 0.05).

To further investigate the mechanism of* Manilkara zapota* leaf methanol extract induced apoptosis in HeLa cells, we evaluated the transcriptional activity of* cytochrome c* using real-time polymerase chain reaction (PCR). The cells were exposed to different concentrations of* Manilkara zapota *leaf methanol extract (12, 24, and 48 *μ*g/mL) and the release of* cytochrome c *was assessed. As shown in Figure 9, treatment with 24 and 48 *μ*g/mL of* Manilkara zapota *leaf methanol extract resulted in the elevation of* cytochrome c* mRNA level. An earlier study has demonstrated that translocation of Bax to mitochondria can induce the outer mitochondrial membrane potential and thus release of* cytochrome c* to the cytosol [36] which activates caspase cascade and cause apoptotic cell death. In the present study, it is conceivable that sufficient Bax appears to reside at the mitochondrial membrane to trigger* cytochrome c *release after* Manilkara zapota *leaf methanol extract treatment. One of the predominant consequences of mitochondrial* cytochrome c* release is the activation of caspase-3. Among the family of caspases, caspase-3 has been demonstrated as the most often triggered caspase protease in apoptotic cells, which implies its critical role in the apoptotic cell death [37]. Based on the findings, caspase-3 was activated after treatment with* Manilkara zapota *leaf methanol extract treatment and thus triggers the release of* cytochrome c*, suggesting a caspase-dependent signal transduction pathway.

### 3.11. Manilkara Zapota Leaf Methanol Extract Inhibits Activation of EGFR in HeLa Cells

Although growth factor-induced epidermal growth factor receptor (EGFR) signaling is required for several morphogenic processes and involved in many cellular responses, the deregulation of EGFR has been associated with the proliferation and development of cervical cancer [38]. To gain a better understanding in which the* Manilkara zapota *leaf methanol extract induces apoptosis, we checked the changes of the transcriptional activity of EGFR following exposure of* Manilkara zapota *leaf methanol extract (12, 24, and 48 *μ*g/mL) on HeLa cells using quantitative real-time PCR. As illustrated in Figure 9, our data showed that untreated HeLa cells had the highest* EGFR *mRNA levels.* EGFR *expression was significantly reduced in HeLa cells treated with* Manilkara zapota *leaf methanol extract compared to the untreated cells (*P* < 0.05). These data revealed that treatment with* Manilkara zapota* leaf methanol extract could diminish the* EGFR *activation, with the maximum effect observed at a concentration of 48 *μ*g/mL* Manilkara zapota* leaf methanol extract. In addition to the effects observed in* EGFR* activity, the role of NF-*κ*B in the inhibition of HeLa cells elicited by* Manilkara zapota *leaf methanol extract remains elusive. Therefore, we further explored the chemoprevention mechanism of NF-*κ*B on* Manilkara zapota *leaf methanol extract in HeLa cells.

### 3.12. Manilkara Zapota Leaf Methanol Extract Diminishes NF-*κ*B in HeLa Cells

Nuclear factor-kappa B (NF-*κ*B) has been linked with chronic inflammation and cancer [39]. A study demonstrated that constitutively activation of NF-*κ*B is associated with cervical cancer [40]. Hence, the gene expression level of NF-*κ*B in response to different concentrations of* Manilkara zapota *leaf methanol extract (12, 24, and 48 *μ*g/mL) was assessed in HeLa cells. The NF-*κ*B family is a group of inducible transcriptions which is involved in inflammatory and immune responses and thereby inhibited cell apoptosis. An earlier study demonstrated that cancer cells with activated NF-*κ*B are resistant to ionizing radiation and chemotherapeutics. Conversely, inhibition of NF-*κ*B activity markedly elevates the sensitivity of cells to chemotherapeutic agents [41]. The overall analysis indicated that untreated HeLa cells presented the highest NF-*κ*B expression compared with the groups treated with* Manilkara zapota *leaf methanol extract. In* Manilkara zapota *leaf methanol extract treated groups, the phosphorylation and degradation of NF-*κ*B expression were increased in a dose-dependent manner (Figure 9). The suppression of NF-*κ*B transcriptional activity resulting from the treatment of* Manilkara zapota *leaf methanol extract was consistent with the study reported by Tan et al. [42], who showed that brewers' rice inhibited NF-*κ*B expression and thereby activated anti-inflammatory and antioxidant responses. Collectively, the findings presented in this study suggest that* Manilkara zapota *leaf methanol extract may modulate the inhibitory activity of HeLa cells via NF-*κ*B signaling.

**Figure 10:**
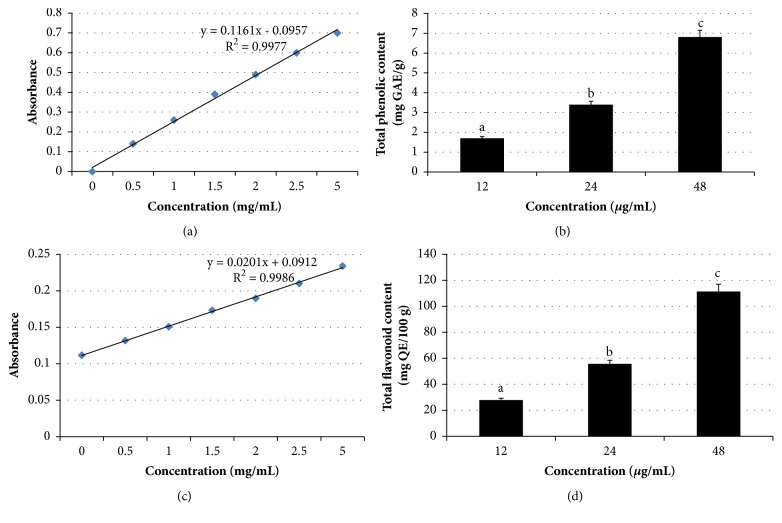
Standard curves of (a) gallic acid and (c) quercetin. Total phenolic (b) and total flavonoid (d) contents in three different concentrations (12, 24, and 48 *μ*g/mL) of* Manilkara zapota *leaf methanol extract. Values are reported as mean ± SD (n = 3). Value with different superscript letter indicates significant difference between groups by Tukey's test (*P *< 0.05).

Most studies have demonstrated the synergistic and/or additive protective effects of several constituents [43]. We speculated that this could be partially due to the polyphenolic components, which synergistically contribute to this antiproliferative effect and apoptosis induction. Furthermore, we also found that* Manilkara zapota *leaf methanol extract contains total phenolic (42.55 ± 5.15 mg GAE/g), total flavonoid (11.60 ± 2.12 mg QE/100 g) (Figure 10), and antioxidant activity as determined using *β*-carotene bleaching test (42.94 ± 3.73%) and 1,1-diphenyl-2-picryl-hydrazyl (DPPH) radical scavenging capacity (0.48 ± 0.01 mg/mL), and phenolic compounds [mainly caffeic acid (0.04 ± 0.01 *µ*g/g), vanillic acid (0.93 ± 0.01 *µ*g/g), p-coumaric acid (0.89 ± 0.16 *µ*g/g), and ferulic acid (79.24 ± 15.95 *µ*g/g)] (unpublished data).  Table 3 shows the antioxidant activity and bioactive components in different concentrations of* Manilkara zapota *leaf methanol extract.

Phytochemical screening is one of the methods that have been employed to evaluate the antioxidant constituents in a plant sample. There are three categories of plant chemicals, namely, phenolic metabolites, terpenoids, and alkaloids [44]. Of these plant chemicals, phenolic compounds are the most critical for dietary applications due to an inverse associated with chronic diseases [45]. Emerging evidence had proved their protective activity against human diseases [46, 47]. Of all phytochemicals, only saponins are present in* Manilkara zapota *leaf methanol extract. None of the steroids, triterpenoids, flavonoids, and phlobatannins is detected in the extract. In support of these findings, previous qualitative analysis of* Manilkara zapota *seed methanol extract has exhibited the presence of saponins, glycosides, and phenols [48]. Taken together, the observed apoptotic effect and reduction of* EGFR *and NF-*κ*B transcriptional activities are likely attributed to the synergistic/additive effects of the phenolic compounds and antioxidant activity present in* Manilkara zapota *leaf methanol extract.

## 4. Conclusions

Our data suggest that* Manilkara zapota *leaf methanol extract may have extensive application as an anticervical cancer agent. Importantly, this extract is nontoxic with regard to the cell proliferation of mouse fibroblast (BALB/c 3T3) cell line. Treatment with* Manilkara zapota *leaf methanol extract led to a collapsed mitochondrial membrane potential which subsequently triggered the release of* cytochrome c*, thus leading to the caspase cascade and ultimately resulting in the activation of the mitochondrial pathway which may play a vital role in the apoptosis. Additionally, the decreased viability of HeLa cells via induction of apoptosis and reduction of* EGFR *and NF-*κ*B transcriptional activities is expected to lead* Manilkara zapota *leaf methanol extract on target epithelial cells to suppress the proliferation of cancerous lesions in the context of cancer chemoprevention. However, further studies are warranted to evaluate the anticancer activity of* Manilkara zapota *leaf methanol extract in animal models in order to provide valuable insights to develop it as a therapeutic approach for the treatment of human cervical cancer.

## Figures and Tables

**Table 1:** Nucleotide sequence of PCR primers for amplification and sequence-specific detection of cDNA (obtained from GenBank database).

Primer name [accession number]	Oligonucleotides (5′-3′) sequence
*Cytochrome c* [JF919224.1]	F: ATCACCTTGAAACCGACCTG
	R: CTCCCTGAGGATAACGCAAA
*EGFR* [NM_005228.3]	F: CAGCGCTACCTTGTCATTCA
	R: TGCACTCAGAGAGCTCAGGA
NF-*κ*B [M58603]	F: TGGAAGCACGAATGACAGAG
	R: TGAGGTCCATCTCCTTGGTC
*ACTB* ^a^ [NM_001101.3]	F: AGAGCTACGAGCTGCCTGAC
	R: AGCACTGTGTTGGCGTACAG
*GAPDH* ^a^ [NM_002046.4]	F: GGATTTGGTCGTATTGGGC
	R: TGGAAGATGGTGATGGGATT
18S rRNA^a^ [HQ387008.1]	F: GTAACCCGTTGAACCCCATT
	R: CCATCCAATCGGTAGTAGCG

*ACTB*: beta-actin, *EGFR*: epidermal growth factor receptor, *GAPDH*: glyceraldehyde-3-phosphate dehydrogenase, NF-*κ*B: nuclear factor-kappa B.

^a^Housekeeping gene.

**Table 2:** Treatment of *Manilkara zapota* leaf methanol extract (1.56-200 *μ*g/mL) on selected cancer cell lines for 24, 48, and 72 h evaluated by MTT and LDH assays.

Cancer cell lines		MTT (*μ*g/mL)			LDH (*μ*g/mL)	
	24 h	48 h	72 h	24 h	48 h	72 h
HT-29	93.27 ± 17.19^a^	89.29 ± 6.01^a^	69.12 ± 8.10^b^	90.33 ± 15.79^a^	85.99 ± 4.87^a^	76.22 ± 5.39^b^
HCT-116	90.14 ± 14.23^a^	87.33 ± 9.29^a^	83.17 ± 9.92^a^	93.22 ± 9.03^a^	90.12 ± 9.77^a^	88.11 ± 11.69^a^
HeLa	89.29 ± 18.20^a^	59.23 ± 10.33^a^	23.87 ± 5.02^b^	87.33 ± 14.98^a^	80.44 ± 11.65^a^	25.76 ± 8.93^b^
HGT-1	80.11 ± 10.19^a^	72.04 ± 5.23^a^	49.44 ± 10.62^b^	65.20 ± 14.27^a^	62.11 ± 6.29^a^	59.89 ± 10.27^a^
HepG2	97.29 ± 3.26^a^	83.95 ± 9.20^ab^	73.02 ± 9.33^b^	89.45 ± 16.82^a^	83.03 ± 5.35^a^	77.04 ± 9.93^a^

HCT-116: human colon carcinoma, HeLa: human cervical cancer, HepG2: human hepatocellular carcinoma, HGT-1: human gastric adenocarcinoma, HT-29: human colorectal adenocarcinoma, LDH: lactate dehydrogenase, and MTT: 3-(4,5-dimethylthiazol-2-yl)-2,5-diphenyltetrazolium bromide.

Values are reported as mean ± SD (n = 3). Value with different superscript letter in the same row for their respective assay indicates significant difference by Tukey's test (*P *< 0.05). In MTT assay, treatment with *Manilkara zapota* leaf methanol extract for 72 h (69.12 ± 8.10 *μ*g/mL) significantly inhibited the proliferation of HT-29 cells compared to 24 h (93.27 ± 17.19 *μ*g/mL) (*P *< 0.05), whereas, in LDH assay, there was a significant effect of the cytotoxic activities of *Manilkara zapota* leaf methanol extract in HT-29 cells incubated for 72 h (76.22 ± 5.39 *μ*g/mL) compared to those incubated for 24 h (90.33 ± 15.79 *μ*g/mL) or 48 h (85.99 ± 4.87 *μ*g/mL) (*P *< 0.05).

**Table 3:** Antioxidant activity and bioactive components in different concentrations of *Manilkara zapota *leaf methanol extract.

Antioxidant activity and bioactive components	12 *μ*g/mL	24 *μ*g/mL	48 *μ*g/mL
Beta-carotene bleaching test (%)	2.58 ± 0.78^a^	5.15 ± 1.22^b^	10.31 ± 1.42^c^
1,1-diphenyl-2-picryl-hydrazyl (DPPH) (mg/mL)	0.010 ± 0.005^a^	0.020 ± 0.001^b^	0.050 ± 0.003^c^
Caffeic acid (*μ*g/g)	0.0005 ± 0.0001^a^	0.0010 ± 0.0003^b^	0.0019 ± 0.0002^c^
Vanillic acid (*μ*g/g)	0.010 ± 0.002^a^	0.020 ± 0.001^b^	0.050 ± 0.004^c^
p-coumaric acid (*μ*g/g)	0.11 ± 0.05^a^	0.21 ± 0.01^b^	0.43 ± 0.12^c^
Ferulic acid (*μ*g/g)	0.95 ± 0.12^a^	1.90 ± 0.09^b^	3.80 ± 0.11^c^

Values are reported as mean ± SD (n = 3). Value with different superscript letter in the same row indicates significant difference between groups by Tukey's test (*P *< 0.05).

## Data Availability

All the data are contained within the manuscript.

## References

[1] World Health Organization (2017). *Cervical Cancer*.

[2] Tewari K., Monk B., Disaia P., Creasman W. (2012). Invasive cervical cancer. *Clinical Gynecologic Oncology*.

[3] Wykosky J., Fenton T., Furnari F., Cavenee W. K. (2011). Therapeutic targeting of epidermal growth factor receptor in human cancer: Successes and limitations. *Chinese Journal of Cancer*.

[4] Xia Y., Shen S., Verma I. M. (2014). NF-*κ*B, an active player in human cancers. *Cancer Immunology Research*.

[5] Đurđević S., Šavikin K., Živković J. (2018). Antioxidant and cytotoxic activity of fatty oil isolated by supercritical fluid extraction from microwave pretreated seeds of wild growing Punica granatum L.. *The Journal of Supercritical Fluids*.

[6] Mazewski C., Liang K., Gonzalez de Mejia E. (2018). Comparison of the effect of chemical composition of anthocyanin-rich plant extracts on colon cancer cell proliferation and their potential mechanism of action using in vitro, in silico, and biochemical assays. *Food Chemistry*.

[7] Do Q. D., Angkawijaya A. E., Tran-Nguyen P. L. (2014). Effect of extraction solvent on total phenol content, total flavonoid content, and antioxidant activity of Limnophila aromatic. *Journal of Food and Drug Analysis*.

[8] Chen C., Wang L., Wang R. (2018). Phenolic contents, cellular antioxidant activity and antiproliferative capacity of different varieties of oats. *Food Chemistry*.

[9] Ke Z. L., pan Y., Xu X., Nie C., Zhou Z. (2015). Citrus flavonoids and human cancers. *Journal of Food and Nutrition Research*.

[10] Rajendran P., Nandakumar N., Rengarajan T. (2014). Antioxidants and human diseases. *Clinica Chimica Acta*.

[11] Ghani A. (2003). *Medicinal Plants of Bangladesh: Chemical Constituents and Uses*.

[12] Haji Mohiddin M. Y., Chin W., Holdsworth D. (1992). Traditional medicinal plants of Brunei Darussalam part III. Sengkurong. *International Journal of Pharmacognosy*.

[13] Tan B. L., Norhaizan M. E., Suhaniza H. J., Lai C. C., Norazalina S., Roselina K. (2013). Antioxidant properties and antiproliferative effect of brewers’ rice extract (temukut) on selected cancer cell lines. *International Food Research Journal*.

[14] Aebi H. (1984). Catalase in vitro. *Methods in Enzymology*.

[15] Meda A., Lamien C. E., Romito M., Millogo J., Nacoulma O. G. (2005). Determination of the total phenolic, flavonoid and proline contents in Burkina Fasan honey, as well as their radical scavenging activity. *Food Chemistry*.

[16] Shanmugapriya K., Saravana P. S., Payal H., Peer Mohammed S., Binnie W. (2011). Antioxidant activity, total phenolic and flavonoid contents of Artocarpus heterophyllus and Manilkara zapota seeds and its reduction potential. *International Journal of Pharmacy and Pharmaceutical Sciences*.

[17] Zhang D., Hamauzu Y. (2004). Phenolics, ascorbic acid, carotenoids and antioxidant activity of broccoli and their changes during conventional and microwave cooking. *Food Chemistry*.

[18] Tan B. L., Norhaizan M. E., Yeap S. K., Roselina K. (2015). Water extract of Brewers' rice induces antiproliferation of human colorectal cancer (HT-29) cell lines via the induction of apoptosis. *European Review for Medical and Pharmacological Sciences*.

[19] Harborne J. B. (1973). *Phytochemical Methods*.

[20] Evans W. C. (1997). *Treasae and Evans Pharmacognosy*.

[21] Dai J., Mumper R. J. (2010). Plant phenolics: extraction, analysis and their antioxidant and anticancer properties. *Molecules*.

[22] Prayong P., Barusrux S., Weerapreeyakul N. (2008). Cytotoxic activity screening of some indigenous Thai plants. *Fitoterapia*.

[23] Zhang N., Bing T., Liu X. (2015). Cytotoxicity of guanine-based degradation products contributes to the antiproliferative activity of guanine-rich oligonucleotides. *Chemical Science*.

[24] Bhat T. A., Chaudhary A. K., Kumar S. (2017). Endoplasmic reticulum-mediated unfolded protein response and mitochondrial apoptosis in cancer. *Biochimica et Biophysica Acta (BBA) - Reviews on Cancer*.

[25] Zou Z.-Z., Nie P.-P., Li Y.-W. (2017). Synergistic induction of apoptosis by salinomycin and gefitinib through lysosomal and mitochondrial dependent pathway overcomes gefitinib resistance in colorectal cancer. *Oncotarget*.

[26] Hsu Y.-C., Chiang J.-H., Yu C.-S. (2017). Antitumor effects of deguelin on H460 human lung cancer cells in vitro and in vivo: Roles of apoptotic cell death and H460 tumor xenografts model. *Environmental Toxicology*.

[27] Zhu Y., Tchkonia T., Fuhrmann-Stroissnigg H. (2016). Identification of a novel senolytic agent, navitoclax, targeting the Bcl-2 family of anti-apoptotic factors. *Aging Cell*.

[28] Nieves-Neira W., Pommier Y. (1999). Apoptotic response to camptothecin and 7-hydroxystaurosporine (UCN-01) in the 8 human breast cancer cell lines of the NCI anticancer drug screen: multifactorial relationships with topoisomerase I, protein kinase C, Bcl-2, p53, MDM-2 and caspase pathways. *International Journal of Cancer*.

[29] Victorino V. J., Pizzatti L., Michelletti P., Panis C. (2014). Oxidative stress, redox signaling and cancer chemoresistance: putting together the pieces of the puzzle. *Current Medicinal Chemistry*.

[30] Tsuchiya A., Kaku Y., Nakano T., Nishizaki T. (2015). Diarachidonoylphosphoethanolamine induces apoptosis of malignant pleural mesothelioma cells through a Trx/ASK1/p38 MAPK pathway. *Journal of Pharmacological Sciences*.

[31] Du J., He D., Sun L.-N. (2010). Semen Hoveniae extract protects against acute alcohol-induced liver injury in mice. *Pharmaceutical Biology*.

[32] Zou X., Liang J., Sun J. (2016). Allicin sensitizes hepatocellular cancer cells to anti-tumor activity of 5-fluorouracil through ROS-mediated mitochondrial pathway. *Journal of Pharmacological Sciences*.

[33] Goyal M. M., Basak A. (2010). Human catalase: Looking for complete identity. *Protein & Cell*.

[34] Yeh C.-C., Tseng C.-N., Yang J.-I. (2012). Antiproliferation and induction of apoptosis in Ca9-22 oral cancer cells by ethanolic extract of *Gracilaria tenuistipitata*. *Molecules*.

[35] Aydos O. S., Avci A., Özkan T. (2011). Antiproliferative, apoptotic and antioxidant activities of wheatgrass (Triticum aestivum L.) extract on CML (K562) cell line. *Turkish Journal of Medical Sciences*.

[36] Goping I. S., Gross A., Lavoie J. N. (1998). Regulated targeting of BAX to mitochondria. *The Journal of Cell Biology*.

[37] Kim H., Choi H., Lee S. K. (2016). Epstein-Barr virus microRNA miR-BART20-5p suppresses lytic induction by inhibiting BAD-mediated caspase-3-dependent apoptosis. *Journal of Virology*.

[38] Narayanan R., Kim H. N., Narayanan N. K., Nargi D., Narayanan B. A. (2012). Epidermal growth factor-stimulated human cervical cancer cell growth is associated with EGFR and cyclin D1 activation, independent of COX-2 expression levels. *International Journal of Oncology*.

[39] Korniluk A., Koper O., Kemona H., Dymicka-Piekarska V. (2017). From inflammation to cancer. *Irish Journal of Medical Science*.

[40] Zhang J., Wu H., Li P., Zhao Y., Liu M., Tang H. (2014). NF-*κ*B-modulated miR-130a targets TNF-*α* in cervical cancer cells. *Journal of Translational Medicine*.

[41] Wang C.-Y., Mayo M. W., Baldwin A. S. (1996). TNF- and cancer therapy-induced apoptosis: potentiation by inhibition of NF-*κ*B. *Science*.

[42] Tan B. L., Norhaizan M. E., Huynh K., Yeap S. K., Hazilawati H., Roselina K. (2015). Brewers' rice modulates oxidative stress in azoxymethane-mediated colon carcinogenesis in rats. *World Journal of Gastroenterology*.

[43] Tan B. L., Norhaizan M. E., Huynh K. (2015). Water extract of brewers' rice induces apoptosis in human colorectal cancer cells via activation of caspase-3 and caspase-8 and downregulates the Wnt/*β*-catenin downstream signaling pathway in brewers' rice-treated rats with azoxymethane-induced colon carcinogenesis. *BMC Complementary and Alternative Medicine*.

[44] Santhosh S. K., Venugopal A., Radhakrishnan M. C. (2016). Study on the phytochemicals, antibacterial and antioxidant activities of Simarouba glauca. *South Indian Journal of Biological Sciences*.

[45] Pereira C., Barros L., Ferreira I. C. (2016). Extraction, identification, fractionation and isolation of phenolic compounds in plants with hepatoprotective effects. *Journal of the Science of Food and Agriculture*.

[46] Freires I. A., De Alencar S. M., Rosalen P. L. (2016). A pharmacological perspective on the use of Brazilian Red Propolis and its isolated compounds against human diseases. *European Journal of Medicinal Chemistry*.

[47] Hamed A. I., Said R. B., Kontek B. (2016). LC-ESI-MS/MS profile of phenolic and glucosinolate compounds in samh flour (Mesembryanthemum forsskalei Hochst. ex Boiss) and the inhibition of oxidative stress by these compounds in human plasma. *Food Research International*.

[48] Mohanapriya C., Uma S., Modilal R. D., Nithyalakshmi V. (2014). Phytochemical screening and in vitro antioxidant studies on acetone extract of Manilkara zapota L. seeds. *International Journal of Pharmaceutical Sciences and Research*.

